# Combining losartan with radiotherapy increases tumor control and inhibits lung metastases from a HER2/neu-positive orthotopic breast cancer model

**DOI:** 10.1186/s13014-021-01775-9

**Published:** 2021-03-04

**Authors:** Wende Li, Sen Li, Ivy X. Chen, Yujiao Liu, Rakesh R. Ramjiawan, Chi-Ho Leung, Leo E. Gerweck, Dai Fukumura, Jay S. Loeffler, Rakesh K. Jain, Dan G. Duda, Peigen Huang

**Affiliations:** 1 Edwin L. Steele Laboratories, Department of Radiation Oncology, Massachusetts General Hospital, Harvard Medical School, 100 Blossom Street, Cox-7, Boston, MA 02114 USA; 2 Present Address: Guangdong Laboratory Animal Monitoring Institute, Guangzhou, 510663 People’s Republic of China; 3 Present Address: Department of Spinal Surgery, Affiliated Traditional Chinese Medicine Hospital, Southwest Medical University, Luzhou, 646000 People’s Republic of China; 4 Present Address: Division of Urology, Department of Surgery, Prince of Wales Hospital, The Chinese University of Hong Kong, Shatin, Hong Kong

**Keywords:** Radiotherapy, Losartan, Angiotensin receptor blocker, Metastatic breast cancer, Mouse model

## Abstract

**Background:**

Patients with metastatic HER2/neu-positive (HER2/neu +) breast cancer (BC) often experience treatment resistance, disease recurrences and metastases. Thus, new approaches for improving the treatment of HER2/neu + BC to prevent metastatic dissemination are urgently needed. Our previous studies have shown that losartan, an angiotensin receptor blocker, increases tumor perfusion and decreases hypoxia in a number of tumor models. Hypoxia reduces the efficacy of radiation and increases metastases. We therefore hypothesized that by modifying tumor stroma and increasing oxygenation, losartan will improve the outcome of radiotherapy and inhibit disease progression in a highly metastatic HER2/neu + murine BC model.

**Methods:**

We established a metastatic HER2/neu + murine BC line (MCa-M3C) and used it to generate mammary fat pad isografts in syngeneic female FVB/N mice. Starting on day 3 after orthotopic tumor implantation, we administered a 7-day losartan treatment (40 mg/kg BW, gavage daily); or a 7-day losartan treatment followed by 20 Gy single dose local irradiation (S-IR) on day 10 (tumor size ~ 100 mm^3^), or 20 Gy local fractionated (5 × 4 Gy daily) irradiation (F-IR) on days 10–14. We analyzed tumor-growth delay (TGD), development of spontaneous lung metastases, animal survival, tumor vascular density, and tumor hypoxia.

**Results:**

Treatments with S-IR, F-IR, Losartan + S-IR, or Losartan + F-IR resulted in a significantly increased TGD (8–16 days) in MCa-M3C tumors versus controls. However, the combination of Losartan + S-IR and Losartan + F-IR further enhanced tumor response to radiation alone by increasing TGD an additional 5 to 8 days for both single and fractionated dose irradiation (*P* < 0.01), decreasing lung metastasis (Losartan + IR *vs.* Control, *P* < 0.025), and increasing animal survival (Losartan + IR *vs.* Control, *P* = 0.0303)*.* In addition, losartan treatment significantly increased tumor vascularity (*P* = 0.0314) and decreased pimonidazole positive (hypoxic) area (*P* = 0.0002).

**Conclusions:**

Combining losartan with local irradiation significantly enhanced tumor response, at least in part via reduced tumor hypoxia presumably due to increased tumor perfusion. Our findings suggest that combining losartan with radiotherapy is a potential new treatment strategy for local control and inhibiting metastasis in HER2 + BC.

**Supplementary information:**

The online version contains supplementary material available at 10.1186/s13014-021-01775-9.

## Introduction

Breast cancer (BC) is the most commonly diagnosed malignancy and the second leading cause of cancer-related death in women worldwide [1–5]. The American Cancer Society (ACS) estimated that in 2020 there will be about 276,480 new invasive BC cases in the United States, and nearly 42,170 of these patients will die from metastatic breast cancer (mBC) (ACS, 2020). BC is a complex and highly heterogeneous malignant disease; approximately 20–30% of BCs have amplification and/or overexpression of the human epidermal growth factor receptor 2 (ERBB2, HER2, HER2/neu, c-erbB-2) oncogenes. Overexpression of HER2/neu or HER2/neu-positive (HER2/neu +) in human BCs indicates an aggressive disease phenotype, and is strongly associated with development of drugs resistance, higher recurrence rates, higher incidence of metastases, and shorter survival [2–10]. HER2/neu amplification promotes tumor cell proliferation and motility in vitro, as well as tumorigenicity and early systemic spread in vivo [10–14]. To treat HER2/neu + BCs, the anti-HER2 humanized monoclonal antibodies pertuzumab plus trastuzumab, and the HER2 tyrosine kinase inhibitors lapatinib, neratinib or tucatinib, are widely and routinely used in clincal practice [15–18]. Together with chemotherapy and/or radiotherapy, these anti-HER2 molecular targeting agents have demonstrated significant control of primary HER2/neu + BCs, resulting in better survival outcomes [16–20]. However, about 25% of early HER2/neu + BC patients still experience disease recurrence after initial anti-HER2 therapy. Moreoever, some patients may show no response or develop drug resistance after a period of anti-HER2 therapy. Eventually these agents are unable to prevent dissemination of all metastatic cancer cells leading to metastatic disease and the death of a subset of BC patients, who initially benefited from the treatment [14–22]. Thus, additional strategies are desperately needed to improve the response of HER2/neu + BC to multimodel treatments. Unfortunately, highly metastatic preclinical models of HER2/neu + BC are scarce, making preclinical development of such therapies a challenge. Therefore, establishement of a novel and clincally relevant model is urgently needed [21–25].

We have established a highly metastatic HER2/neu + mammary tumor line (MCa-M3C) from the MMTV-PyVT/FVB transgenic mouse, which developed spontaneous mammary tumors. This metastatic MCa-M3C HER2/neu + tumor has a highly desmoplastic stroma, mimics the clinical feature of difficult-to-treat BCs, is ER + , and has served as an ideal model to study new therapeutic strategies and dissect the underlying mechanisms of response to treatment in our laboratory [26–28].

We previously found that in highly desmoplastic malignant cancers such as breast and pancreatic carcinomas, components of the tumor microenvironment including cancer cells, stromal cells, and the fibrotic extracellular matrix (ECM) actively contribute to buildup of solid stress, which confers treatment resistance by collapsing the tumor vessels [29–31, 53]. As tumor blood vessels are structurally abnormal, and easily collapsible under this high compressive force, the resulting reduction in tumor perfusion increases tumor hypoxia [29, 31]. Tumoral hypoxia has been shown to promote more aggressive phenotypes, immunosuppression, and eventually leads to resistance to chemo/radiotherapy and immunotherapy [32, 33]. The cellular components store and transmit solid stress through the interstitial matrix molecules collagen and hyaluronan, making both cancer-associated fibroblasts (CAFs) and ECM critical targets for the decompression of tumor vessels [29, 32, 34, 35]. We recently reported that losartan—a drug commonly used in the clinic to treat hypertension—improves treatment outcome by decompressing collapsed tumor blood vessels, and thus increasing tumor perfusion in several murine cancer models [29, 33, 36, 37]. Losartan through its activity to block angiotensin II receptor type 1 (AT1), could decrease the intra-tumoral expression of thrombospondin-1 (TNBS-1), and significantly reduce tumor collagen and hyaluronan production [29, 33, 36]. We therefore hypothesized that by modifying tumor stroma and increasing oxygenation, losartan will improve the outcome of radiotherapy and decrease metastases in a highly metastatic MCa-M3C HER2/neu + murine BC model.

Here, we evaluate the effect of combining losartan and radiation in the MCa-M3C orthotopic HER2/neu + BC model by measuring local tumor response, distant metastases, host survival, tumor vascularity and tumor hypoxia. Our results show that combining losartan and single dose or multi-fraction irradiation not only significantly enhances tumor-gowth delay (TGD), but also decreases lung metastasis, and prolongs the overall survival of host mice. Lastly, we show that losartan significantly increases MCa-M3C tumor vascular volume and reduces tumor hypoxia.

## Materials and methods

### Tumor cell line establishment and in vitro doubling time assay

To establish the HER2/neu + MCa-M3C cell line used in this study, we first transplanted a spontaneous mammary adenocarcinoma arising in a female MMTV-PyVT/FVB transgenic mouse [38], into the mammary fat pad (MFP) of syngeneic FVB/N mice [39]. Subsequently, the BC tumor tissues obtained from metastatic lung foci in the FVB/N hosts were re-implanted into the MFP of new recipients. Following 3 serial in vivo selections of BC metastases to the lungs from MFP-implantation, fresh metastatic tumor tissue in the lung was obtained and successfully cultured in vitro as a long-term passage monolayer cell line—named MCa-M3C. For primary cell culture, fresh lung metastatic tumor tissue was quickly cut into small pieces, cleared of necrotic tissue, washed twice in PBS, finely minced, and placed in a 25 cm^2^ (T25) culture flask containing 5 ml of Dulbecco’s modified Eagle’s medium (DMEM) supplemented with 15% heat-inactivated fetal bovine serum (FBS; Gibco, Carlsbad, CA, USA), penicillin (100 units/ml), and streptomycin (100 µg/ml). The cells were incubated in 5% CO_2_ at 37 °C and passaged once or twice weekly to maintain an exponentially growing monolayer [40]. A single cell suspension was prepared, and 5 × 10^4^ cells were plated in T25 flasks in duplicate for an assessment of the cell doubling time (CDT). The cells were trypsinized and counted after 72 h. The CDT was calculated by the equation: CDT = (0.693 × T)/In (N–N_0_); where T is 72 h, N_0_ is the number of cells plated at day 0, and N is the number of cells obtained 72 h later.

### In vitro colony formation assay

For radiation effects, single cell suspensions were prepared, counted, plated on T25 culture flasks, and irradiated 20–24 h later with 320-kV, 12.5 mA, X-ray at a nominal dose rate of 1.67 Gy (Gy) per minute (6 doses: 0 Gy to 10 Gy in 2 Gy increments). Cells were then incubated for 12–16 days for colony formation depending on the dose administered. The surviving fraction (SF) data was corrected for initial and final multiplicity determined 4 to 6 h after plating and at the were time of irradiation [40]. At each dose point, 5 flasks containing a 2–fourfold range of test cells were plated with the intent of obtaining 20–100 colonies/flask. Following irradiation, the cells were routinely cultured for 12 (for control cells) to16 days (for cells exposed to high doses of radiation), i.e., until the number of colonies per flask containing 50 cells did not change. The colonies were then fixed, stained, and counted. The cell SFs were calculated by the formula: SF = (number of colonies/number of cells plated)/PE, where PE equals the fraction of plated cells which form colonies in the absence of radiation. For losartan toxicity effects, 100 cells of a single cell suspension were plated and cultured in DMEM supplemented with 15% FBS and different concentrations (0, 5, 10, 20 µMol/L) of losartan for 12 to14 days; and the number of colonies was counted. All in vitro experiments were repeated at least three times.

### MTT cell viability assay

MCa-M3C cells were seeded in 96 well plates at 2000 cells/well and incubated for 24 h before losartan treatment was initiated. When treatments were completed, 20 µl of 3-(4,5-dimethylthiazol-2-yl)-2,5-diphenyltetrazolium bromide (MTT) reagent (5 mg/ml, Sigma-Aldrich) was added to each well and incubated at 37 °C for 2 h. Then the medium was discarded and 100 ml DMSO (Sigma-Aldrich) was added to each well and incubated for 30 min at room temperature. Absorbance was measured at a wavelength of 550 nm by Benchmark Plus microplate reader (Bio-Rad, USA).

### Animal and in vivo tumor growth

Six to eight-week-old female FVB/N mice were used in our study. All mice were bred and maintained in our Cox-7 defined flora animal facility [39], and experimental mice were housed in micro-isolator cages, fed with sterile laboratory pellets, and given acidified sterile water ad libitum. All animal care and experiment related procedures were carried out following the *Public Health Service Policy on Humane Care of Laboratory Animals* and approved by the Institutional Animal Care and Use Committee at the Massachusetts General Hospital.

To generate MCa-M3C source tumors for the current study (which were initiated from in vitro cell cultures), and to evaluate the cells’ tumorigenicity (tumor “take rate”): approximately 10^5^ tumor cells (in 0.5 ml PBS) were injected into the third MFP of FVB/N female mice. Following tumor cells injection, mice were observed weekly for tumor development and growth up to eight weeks post-injection. The MFP tumors that grew beyond 3 mm in diameter and showed progressive growth were counted as tumor “takes”. Then a suspension of a MCa-M3C tumor grown to ~ 10 mm in diameter was transplanted serially into new FVB/N mice by orthotopic MFP implantation for up to 4 passage (F4) to continuously monitor tumor transplantability.

### Evaluation of orthotopic tumor responses to combined losartan and radiotherapy

Small (1–2 mm diameter) MCa-M3C source tumor fragments (in vivo F2 to F4 passages of isografts) were implanted into the third MFP of FVB/N female mice. A single source tumor from each passage was used to initiate all tumors for each experimental cohort. After implantation, two perpendicular tumor diameters were measured once to twice per week with calipers. Tumor volume (*V*) was calculated as *V* = *a* x *b*^2^/2, where *a* and *b* are the long and short axes, respectively [39]. At day 3 after orthotopic tumor implantation, the experimental mice received: (i) a 7-day treatment with losartan, 40 mg/kg body weight (BW) once per day by oral gavage; (ii) a 7-day treatment with losartan, 40 mg/kg BW gavaged daily, followed by either a 20 Gy single dose local irradiation (S-IR) at day 10 (tumor at ~ 6 × 6 mm diameter); or 20 Gy in 5 fractions (4 Gy, daily for 5 days) local irradiation (F-IR) on day 10 to14; (iii) radiation alone (S-IR or F-IR for a total dose of 20 Gy); and (iv) sterile water 0.2 ml gavaged daily for 7 days as normal control. Single or multi-fraction (20 Gy) irradiation (320-kV, 12.5 mA, 3.76 Gy per minute) was given under normal blood flow conditions utilizing a 1 cm field centered over the MFP tumor; followed by determination of the tumor-growth delay (TGD).

### Metastasis and survival assays

To determine metastatic frequency, the isografted orthotopic tumors implanted in the third MFP were surgically resected when the tumors reached a size of 10 × 10 mm (or approximately 500 mm^3^). The tumor bearing mice were anesthetized (90 mg/kg ketamine and 9 mg/kg xylazine, i.p.), and the MFP tumor-covering skin was sterilized using 70% alcohol. A skin incision was made to expose the MFP tumor mass, and intact tumor nodules were removed by surgical separation/resection from surrounding tissues. Any bleeding was controlled, and the resulting wound was closed with a 9-mm wound autoclips (Clay Adams, Division of Becton Dickinson and Co., Parsippany, NJ). The wound clips were removed 1–2 weeks later. Post-operative analgesia was supplied by administering buprenorphine (0.1 mg/kg s.c. q 12 h) for 3 days. The first dose of buprenorphine was administered about 30 min pre-procedurally as a pre-emptive analgesia for post-operative pain control. Experimental mice undergoing such a procedure could fully reach a normal active condition at 3–7 days post-operation. The mice were euthanized 4 months after primary tumor implantation or when they became moribund, whichever came first. Gross autopsy and pathological analyses were performed on all euthanized mice. Survival experiments were terminated when mice became moribund, or lost more than 15% of body weight, or reached the endpoint at day 120 of tumor implantation.

### In vivo imaging of orthotopic MFP implanted tumors

To observe MCa-M3C orthotopic tumor growth and tumor microvasculature responses to losartan treatment in vivo, the MFP window model and multi-photon laser scanning microscopy (MPLSM) were used [41–43]. These tools permitted intravital imaging of orthotopically grown MCa-M3C tumors and their blood vessels change in real-time through a glass window placed on the MFP tumors [41, 42]. In brief, for making the MFP window, FVB/N mice were anesthetized, and surgery was performed under sterile conditions. A flap of skin (15-mm diameter) from an opposing surface of the MFP skin fold was removed to leave a fascial plane consisting of epidermis with nipple, MFP, and vasculature. Then a fresh piece of MCa-M3C source tumor tissue (1 mm^3^) was implanted into the remaining fascial layer, and a sterile glass coverslip was placed over the center of a frame to cover the surgical area [41]. At day 3 post tumor implantation, the host mice received losartan treatment (40 mg/kg BW gavaged daily), then the in vivo imaging was performed  with MPLSM [44]. The MPLSM was conducted on a custom-built imaging system consisting of a confocal laser-scanning microscope body (BX-51, Optical Analysis) and a femtosecond laser source (High Performance Mai Tai, Spectra-Physics) [42]. Imaging studies were performed at 20 × magnification, with a 0.95 NA water immersion objective (Olympus XLUMPlanFI, 1-UB965, Optical Analysis). Prior to each imaging session, mice were anesthetized, their tail vein was injected with 100 µl rhodamine-dextran (Sigma-Aldrich), and the mice were positioned on a stereotactic frame. For each tumor, four adjacent images were obtained through the MFP window at day 0, and the same regions were revisited and recorded at day 3 post losartan treatment. The percentage of tumor vessels was analyzed by Image J software [42, 43].

### Histopathology and immunohistochemistry

Tumor tissues and all grossly observed lung lesions were collected for histopathologic study. Tissue specimens were fixed in 10% neutral buffered formalin, embedded in paraffin, cut at 5-µm thickness, and routinely stained with hematoxylin and eosin (H&E) for histologic examination. Tumor sections were also stained with Masson’s Trichrome stain for detection of tumor collagen. For evaluation of tumor vascularity, frozen tumor Sects. (7–8 µm thick) were immunostained with primary anti-CD31 (for endothelial cells) antibody and counter stained with DAPI. Samples were imaged by using an Olympus confocal microscope. Pimonidazole (Hypoxyprobe; Hypoxyprobe, Inc.) was used as a marker of tumor hypoxia. Hypoxyprobe (60 mg/kg BW) was injected i.p. 30 min before the mice were euthanized. Tumors were harvested and processed immediately. Hypoxia was assessed in frozen tissue sections by immunofluorescence (IF) staining of pimonidazole by anti-Hypoxyprobe-fluorescein isothiocyanate (FITC)-labeled antibody, as described [41, 44].

### Western blotting

Western blot analysis was performed with 8% SDS/PAGE using standard methods [42]. In brief, MCa-M3C cells were placed directly into RIPA lysis buffer (Boston BioProducts Inc, MA Cat# BP-115) containing Complete ULTRA tablets, Mini EDTA-free (Roche Diagnostics, IN Cat# 05892791001), and PhosStop (Roche Diagnostics, IN Cat# 04906837001), incubated at 4 °C for 15 min, and then centrifuged at 14,000 rpm (~ 18,000Xg), 4 °C for 10 min for protein extraction. Approximately 20 µg of denatured protein per sample lysates were loaded on SDS/PAGE, separated by electrophoresis and transferred onto a polyvinylidene difluoride membrane (PVDF) using wet electroblotting transfer at 200 mA for 2.5 h. Non-specific binding was blocked with 5% non-fat milk for 1 h. Membranes were incubated overnight at 4 °C with the specific primary antibodies, followed by peroxidase-conjugated anti-rabbit HRP secondary antibodies. The anti-p-HER2 (1:1000, Cell Signaling Technology, Danvers, MA Cat#2245S), anti-HER2 (1:1000, Cell Signaling Technology, Danvers, MA Cat#2242S), and β-Actin (1:5000, Sigma, MO Cat#A5441) primary rabbit antibodies were used in the study. Image J software was used for densitometric analysis [42, 45].

### Statistical analysis

Unless otherwise noted, data are presented as mean ± SEM. The one-way ANOVA method followed by the Dunnett’s post hoc test, and the Unpaired t test (two-tailed) was used to compare tumor volumes and the days of TGD of the treatment groups against the control. Difference in tumor metastatic rates were analyzed by Kendall’s tau with the Holm-Bonferroni adjustment. Host survival times (curves) were compared with the Gehan-Breslow-Wilcoxon test. All statistical analyses were performed using GraphPad Prism 7 and SPSS version 24. A difference was considered statistically significant when the *P* value was less than 0.05.

## Results

### Establishment and characterization of MCa-M3C HER2/neu + metastatic mammary tumor model in vitro and in vivo

**Fig. 1:**
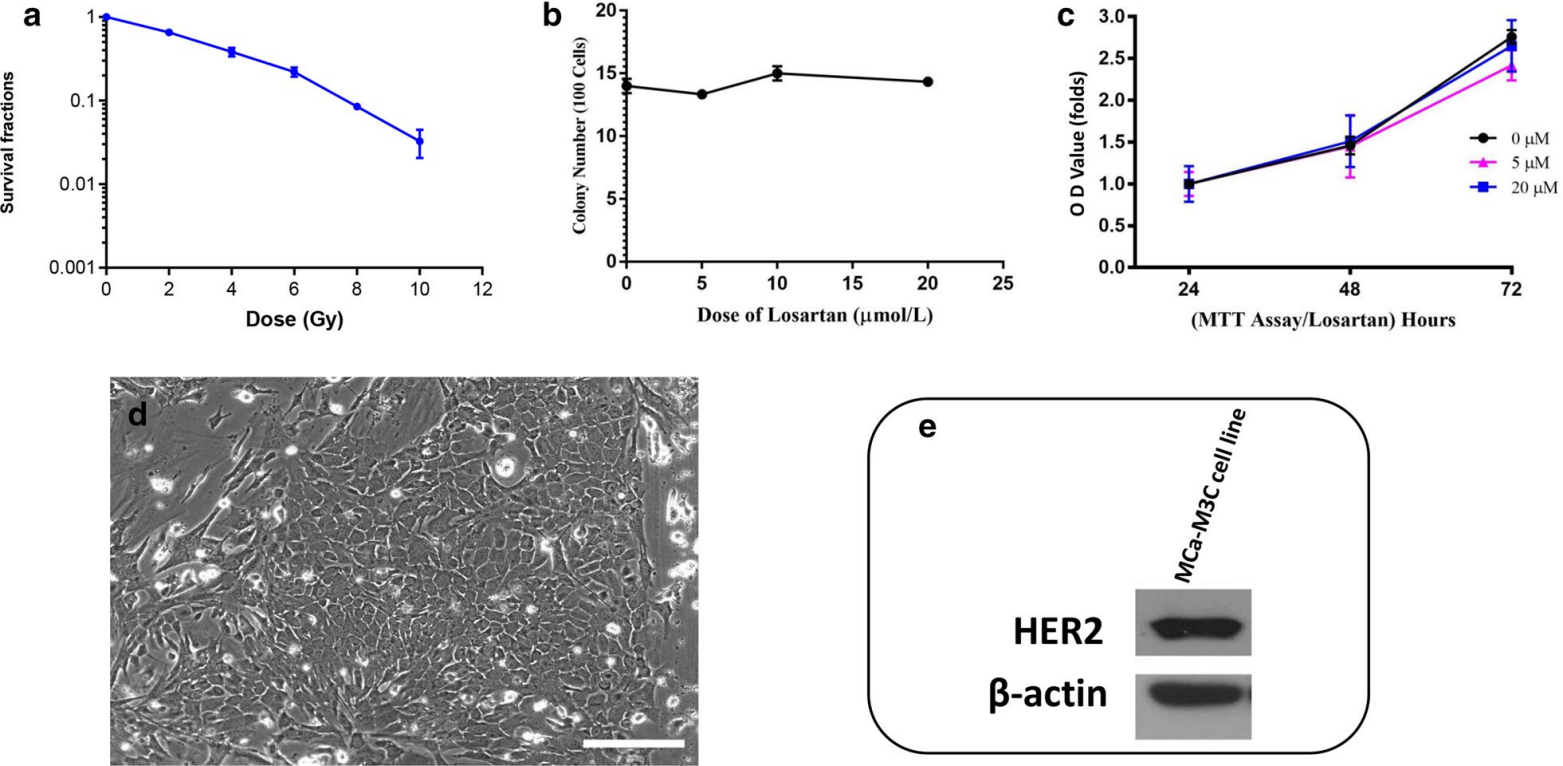
MCa-M3C cell response to treatment, morphological features, and HER2/neu expression. Colony formation assay for **a** MCa-M3C cell response to irradiation, and **b** MCa-M3C cell response to losartan. **c** The absence of an effect of losartan alone on MCa-M3C cell proliferation at doses up to 20 µmol/L. **d** Morphological features of MCa-M3C cells in vitro. The large number of polygonal and a few spindle-shaped cells are seen under the phase contrast microscopy. *Bar* = 100 µm. **e** HER2/neu expression in MCa-M3C cells in vitro by Western blot

**Fig. 2:**
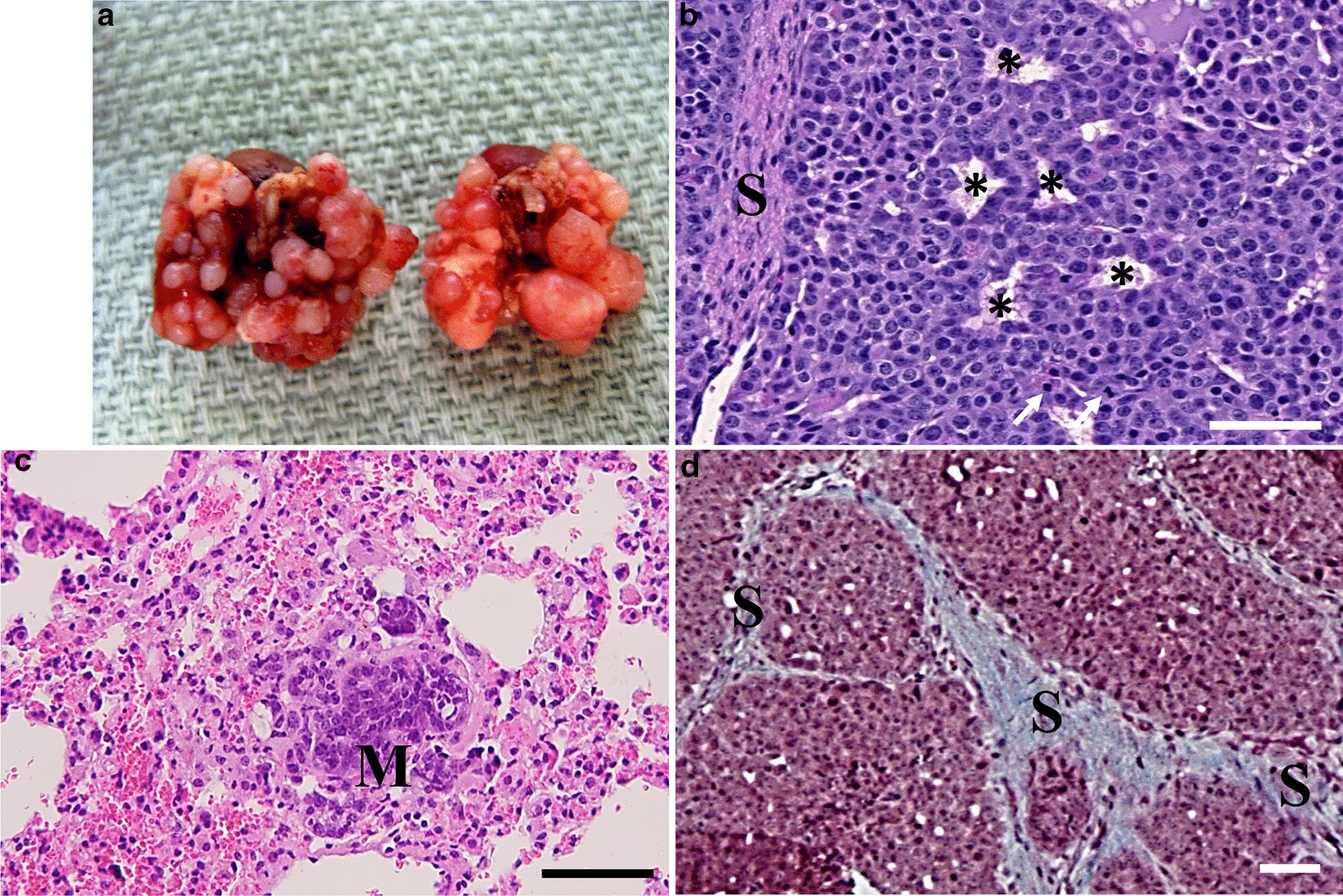
MCa-M3C tumor histological features and lung metastases. **a** Spontaneous lung metastases from MCa-M3C tumor implanted in FVB/N mouse MFP. **b** Photomicrograph of the section of MCa-M3C MFP tumor isograft showing adenocarcinoma histological features, with a glandular-epithelial growth patterns (*); abundant tumor stroma (S); and tumor cell mitotic figures (arrows). H&E stain; *Bar* = 100 µm. **c** Photomicrograph of a section of MCa-M3C metastasis in lung (M), showing tumor cells and the growth pattern similar to that in the primary MFP tumor. H&E stain; *Bar* = 100 µm. **d**  Masson’s Trichrome staining of MCa-M3C MFP tumor isograft reveals a high collagen density (S, positive light blue stain) *Bar* = 100 µm

MCa-M3C cells were established from serial metastases of a spontaneous tumor arising in MMTV-PyVT/FVB mouse as illustrated in an Additional file 1: Fig. 1. In the present study, source tumors were initiated from in vitro monolayer cultures of the cells (Fig. 1d). The cells exhibited a stable CDT (25.3 ± 4.4 h), high PE (0.43 ± 0.04), and resistance to radiation by the colony formation assay in vitro when compared to most other cultured cell types previously reported [36] (Fig. 1a). Neither the colony formation assay nor the MTT assay revealed any significant cytotoxicity of losartan alone (Fig. 1b, c). In vivo, MCa-M3C cells showed high tumorigenicity (100% tumor take rate with 10^5^ tumor cells MFP injection), high tumor transplantability (100%, passage 2–4 of MFP transplantations), and a high frequency of spontaneous metastases (80–100% lung metastases were detected in the host mice at the time the mice became moribund or at 4 months after removal of the primary MFP tumor grafts at a size of ~ 500 mm^3^) (Fig. 2a). Histological examination showed MCa-M3C MFP tumor isografts and their pulmonary metastases retained the histological features of adenocarcinomas (Fig. 2b, c). In addition, these tumors exhibited a high density of collagen as revealed by Masson’s Trichrome staining (Fig. 2d). Furthermore, MCa-M3C cells showed strong HER2/neu + expression by Western blotting (Fig. 1e).

### Combining losartan with radiotherapy significantly increases tumor-growth delay of orthotopic HER2/neu + *MCa-M3C tumors*

**Fig. 3:**
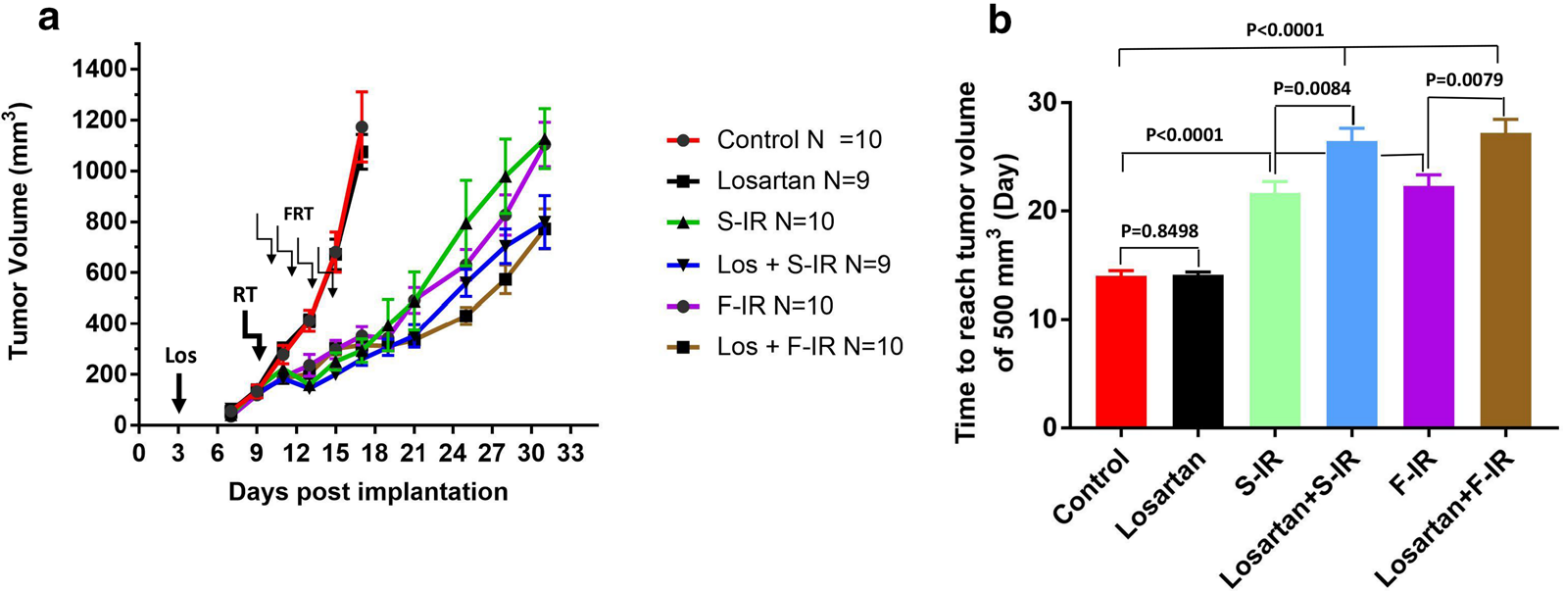
MCa-M3C orthotopic tumor response to combined losartan and single-dose or fractionated dose radiotherapy. **a** MCa-M3C orthotopic tumor growth and response curves of control, losartan alone, 20 Gy local single-dose irradiation alone (S-IR), 20 Gy five fractionated dose irradiation alone (F-IR), combined losartan with single-dose irradiation (Los + S-IR), and losartan with fractionated dose irradiation (Los-F-IR). Los indicates the start losartan treatment; RT indicates single-dose irradiation; and the FRT + arrows indicate the schedules of 5 fraction irradiation treatment. **b** Mean tumor growth time (days) for tumors to reach a mean volume of 500 mm^3^. Results show that S-IR, F-IR, Los + S-IR, or Los + F-IR treatment significantly delays tumors growth for 8 to 16 days compared to control, or losartan alone treatment (Unpaired t test, all *P* < 0.0001; 95% confidence interval between 5.292 to 10.04 and 10.35 to 16.05). In addition, the combination of Los + S-IR, or Los-F-IR treatment significantly enhanced tumor response compared to radiation alone (S-IR or F-IR) by increasing TGD an additional 5 days (Los + S-IR *vs* S-IR, or Los + F-IR *vs* F-IR; Unpaired t test, both *P* < 0.025; 95% confidence interval between 1.405 to 8.150 and 1.457 to 8.343). Losartan alone did not show an anti-tumor effect (Losartan *vs* Control, *P* > 0.05). Data are presented as Mean ± SEM; *P* < 0.05 is considered statistically significant (N = 9–10/group)

To investigate the effect of combining losartan with radiotherapy, we evaluated TGD in the orthotopic HER2/neu MCa-M3C BC model (Fig. 3 & Additional file 1: Fig. 2). We found that treatments of single dose local irradiation (S-IR), or fractionated local irradiation (F-IR), resulted in a substantial TGD, which was further increased by 5 to 8 days when combined with losartan, to reach a tumor volume of 500 mm^3^ (Losartan + S-IR *vs* S-IR, *P* = 0.0084; and Losartan + F-IR *vs* F-IR, *P* = 0.0079). As expected from the in vitro studies (Fig. 1b, c), losartan alone displayed no effect on tumor growth in MCa-M3C orthotopic isografts in vivo.

### Combining losartan with radiotherapy significantly decreases lung metastasis and increases host survival

**Fig. 4:**
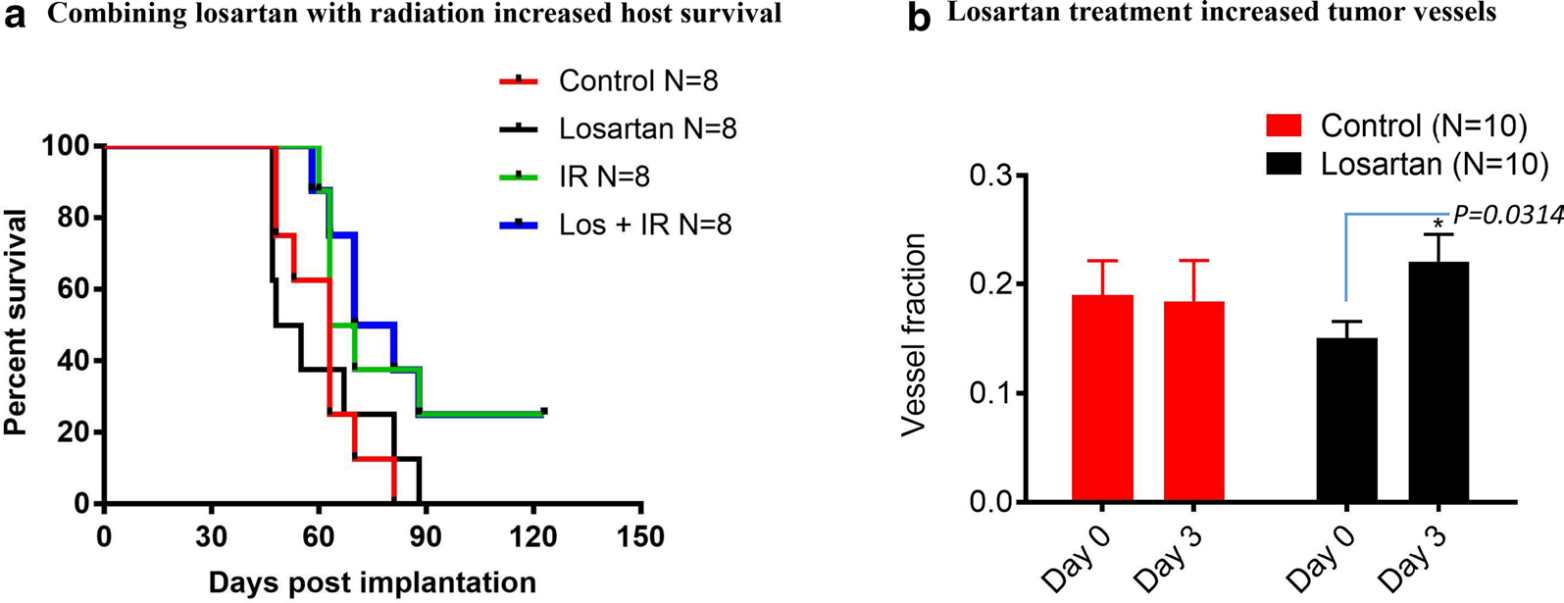
Combining losartan with radiotherapy increased MCa-M3C tumor host survival; and losartan treatment increased tumor vessel fraction. **a** Kaplan–Meier survival curve shows that combining losartan with 20 Gy IR (Los + IR) significantly increased host survival (Los + IR *vs.* Control *P* = 0.0303). **b** Tumor vessels imaging measurement of MCa-M3C tumors growth in MFP windows by MPLSM at day 0 and day 3 post-treatment. Result showed losartan treatment significantly increased tumor vessel fraction (Area of blood vessels/field area, Unpaired t test, *P* = 0.0314)

Next, we evaluated the in vivo efficacy of losartan plus radiation in tumor bearing mice. Both IR alone and Losartan + IR decreased the host metastatic burden compared to controls. However, the decrease was significant only for the combination of Losartan + IR (Losartan + IR *vs.* Control, *P* < 0.025; Table 1, Additional file 1: Fig. 3E). Furthermore, both IR alone and Losartan + IR combination treatment tended to increase host mouse survival when compared to the controls (Fig. 4a). But only the Losartan + IR combination treatment significantly increased overall animal survival (Losartan + IR *vs.* Control, *P* = 0.0303; IR *vs.* Control, *P* = 0.1026). Overall survival after Losartan + IR and IR alone did not significantly differ.

### Losartan treatment increases functional tumor vessels and alleviates tumor hypoxia

**Fig. 5:**
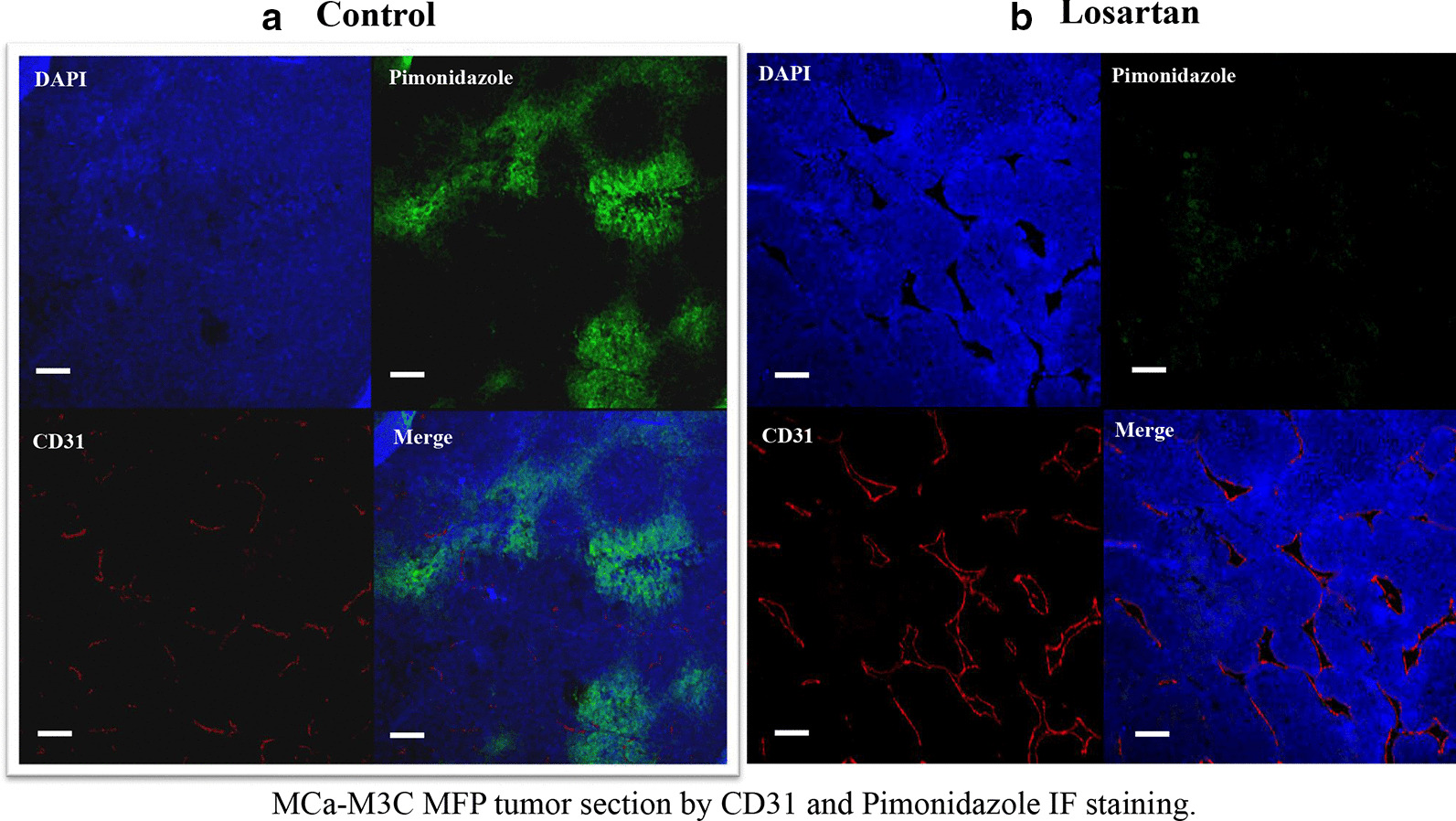
Representative immunohistochemistry of CD31 staining and pimonidazole immunofluorescence (IF) in treated and control MCa-M3C tumor tissues. **a** CD31 and pimonidazole IF staining in the control tumor sections, **b** CD31 and pimonidazole IF staining in losartan-treated tumors. CD31 positive vessels are shown in red and pimonidazole positive hypoxic tissue staining in green. *Bar* = 50 µm

**Fig. 6:**
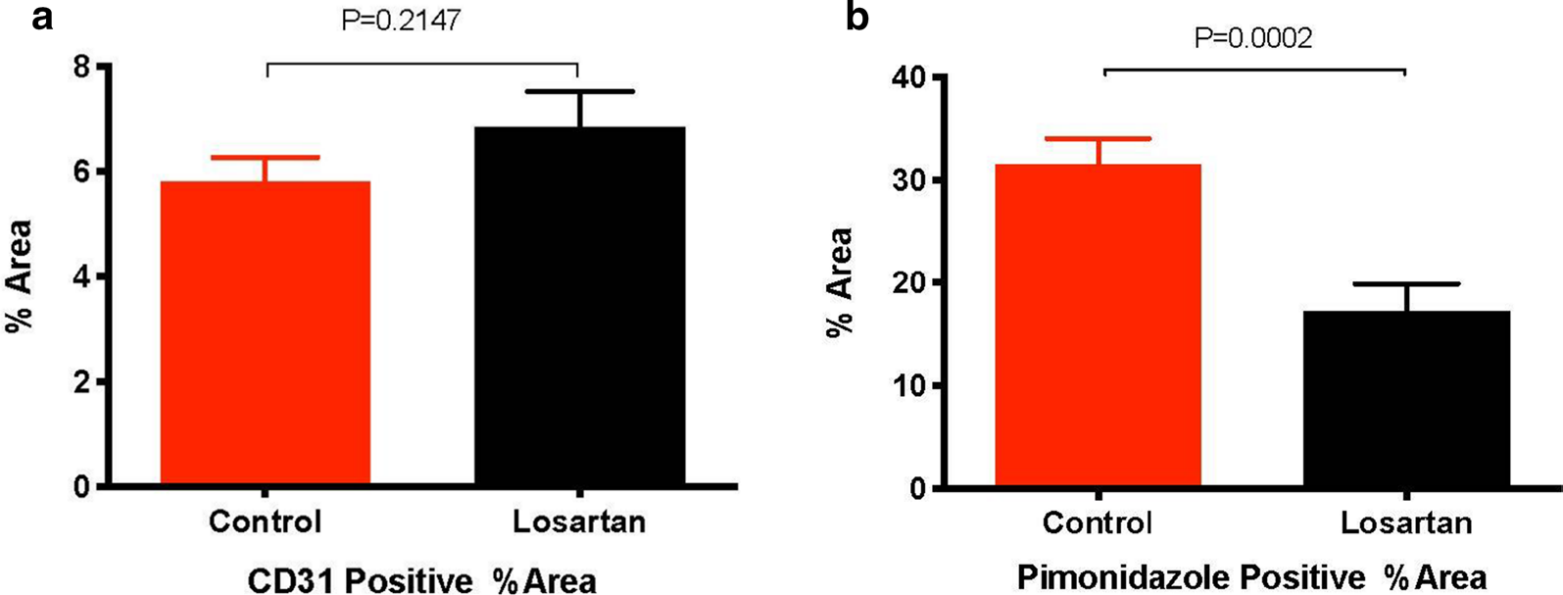
Losartan treatment increased tumor vascular density and significantly decreased tumor hypoxia in MCa-M3C orthotopic tumors. **a** Increases in CD31 positive vascular area after losartan treatment, which is not statistically significant (Losartan *vs.* Control, *P* = 0.2147). **b** Losartan treatment significantly deceases hypoxia measured as pimonidazole positive area (Losartan *vs.* Control, Unpaired t test, *P* = 0.0002) in MCa-M3C tumor tissues (N = 5–10 areas/group)

To evaluate whether losartan improves tumor vessel perfusion, we monitored tumor growth and imaged tumor vascular response to losartan using MPLSM. Changes in the volume of perfused tumor vessels over the course of losartan treatments were analyzed by Image J software (see Methods) (Additional file 1: Fig. 3A-D). Losartan significantly increased the percentage of perfused tumor vessel (vessels fraction—area of blood vessels/field area) at day 3 in the treated tumors (Fig. 4b, *P* = 0.0314), with no significant vascular changes observed in the control tumors. Next, we examined tumor vascular density and tumor hypoxia by CD31 and pimonidazole immunofluorescence staining, in control and losartan treated tumors. We observed a numerically increased CD31 positive tumor vascular density after losartan treatment, but the difference was not statistically significant (*P* = 0.21, Figs. 5, 6a). These changes of tumor vascular density were also observed morphologically in H&E stained  tumor sections (Additional file 1: Fig. 4). The discrepancy between  MPLSM and CD31 staining data is consistent with our previous observation that losartan re-perfuses existing collapsed vessels but does not necessarily generate more blood vessels. However, the treatment significantly decreased the pimonidazole positive (hypoxic) area fraction (*P* = 0.0002; Figs. 5, 6b). Our finding suggests that losartan enhanced tumor response to radiotherapy is associated with increased tumor blood vessels perfusion (tumor vascular volume) and decreased tumor hypoxia.

## Discussion

Clinical treatment of stages I to III BCs usually includes surgery and radiotherapy, often in combination with chemo or other therapies, delivered either before or after surgery (ACS, 2020). The subtype HER2/neu + BC accounts for ~ 25% of all BCs and is associated with a poor prognosis and treatment outcome [2–10]. In the last decades, advancement of multiple anti-HER2 targeted therapies in combination with chemo/radiotherapy have made significant progress in the treatment of HER2/neu + BC. Unfortunately, approximately 30–55% of patients with advanced HER2 + BC eventually develop brain metastases, with associated morbidity and mortality. Furthermore, approximately 70% of HER2/neu + mBC patients (stage IV) eventually develop resistance to HER2-targeted treatment, e.g., with trastuzumab, within one year, and over one third of patients do not respond to this therapy [2–6, 15–18]. Thus, improving treatment of HER2/neu + BC to prevent metastatic dissemination is urgently needed [2–6].

A newly established MCa-M3C HER2/neu + murine BC line has been used in our studies [26–28]. We show here that this cell line exhibits high in vivo tumorigenicity, high orthotopic transplantability in immunocompetent mice, and a high frequency of spontaneous metastasis to the lungs. The MCa-M3C MFP tumors and their pulmonary metastases retain histological features of adenocarcinoma with a high collagen content and dense stroma. Thus, it served as an ideal model to study the combination of losartan with radiotherapy.

This study shows that combining an angiotensin receptor blocker, losartan, with radiotherapy significantly enhances TGD, decrease lung metastasis, and increase host survival in a highly metastatic HER2/neu + MCa-M3C murine BC model. In addition to high collagen density stroma, other bio-pathological characteristics of MCa-M3C make it a very useful model and tool for evaluating clinical therapy response metrics, i.e., local response and growth delay, metastases, and survival *vs* time and the relationship between these metrics [27, 28].

Previous studies have demonstrated that hypoxia and poor blood supply promote an aggressive tumor phenotype and contribute to ineffective systemic anti-cancer chemo-radiotherapy, and treatment resistance. Thus, developing new approaches to prevent and/or alleviate tumor hypoxia has become an active area of research [32, 46–48]. Our previous studies have found that in two orthotopic ovarian cancer models, losartan treatment reduced tumor ECM content, lowered tumor solid stress, improved tumor vessel perfusion and relieved tumor hypoxia. These changes increased delivery of chemotherapeutic drugs, resulting in significantly enhanced chemotherapy efficacy and reduced ascites in tumor-bearing hosts [33]. We also recently reported that losartan combined with the CXCR4 inhibitor AMD3100 could increase the efficacy of radiotherapy in a highly metastatic osteosarcoma mouse model [36]. The therapeutic effects were most likely due to the targeting both of tumor hypoxia and CXCR4 by combining losartan, AMD3100, with local irradiation [36].

It was recently reported by others that losartan could inhibit mammary tumor development and progression to invasive carcinoma [49]; or suppress the growth of pulmonary metastases [50]. In this HER2/neu + MCa-M3C murine BC model, we did not detect a direct growth inhibition after losartan treatment alone on tumor cells in vitro or established tumors in vivo. Instead, we found that losartan treatment significantly increased perfused tumor vessels, and decreased tumor tissue hypoxia in the MCa-M3C MFP implanted isografts. Multi-fraction irradiation (F-IR) did not result in a more pronounced tumor response than single dose irradiation (S-IR), nor did Losartan + F-IR yield a significantly different TGD compared to Losartan + S-IR. This result may be due to many factors, such as tumor cell repopulation rate, tumor tissue hypoxic fraction and reoxygenation, and overall radiation treatment time, all of which may be tumor type dependent [51]. Altogether, our results suggest that losartan can enhance tumor response to radiotherapy by increasing tumor blood perfusion and improving tumor tissue oxygenation.

A proof-of-concept phase II clinical trial of total neoadjuvant therapy with FOLFIRINOX in combination with losartan followed by chemoradiotherapy for locally advanced pancreatic cancer recently showed the potential benefit of adding losartan as a cancer treatment modality [52]. Our results support the clinical testing of losartan with radiotherapy in patients with HER2/neu + BC.

## Conclusion

Using a newly established mouse model of spontaneously metastatic BC with HER2/neu overexpression and a collagen-rich stromal component, we show that combining losartan with single dose or fractionated dose irradiation significantly delays local tumor growth, decreases lung metastases, and increases host survival. These therapeutic effects are most likely due to losartan inducing increased tumor blood perfusion and decreased tumor hypoxia. Our finding suggests that combining losartan with radiotherapy could be a potential new therapeutic strategy for desmoplastic tumors including metastatic HER2 + BC.

**Table 1:** Combining losartan with radiotherapy in HER2/neu-positive metastatic MCa-M3C tumors

Group (N = mice)	Lung macro metastases			
	> = 3 nodules	< 3 nodules;	0 nodule	Chi-square (χ^2^_)_ test
Control N = 8	5	2	1	
Losartan N = 8	3	3	*2*	Los *vs* Control P > 0.05
20 Gy IR N = 8	2	2	*4*	IR w Control P > 0.05
Los + IR N = 8	0	3	5	Los + IR vs control P < 0.025

## Supplementary information

**Additional file 1.** **Figure 1**. Schematic representation of the protocol for MCa-M3C cell line establishment. Tumor tissue was obtained from a spontaneous mammary adenocarcinoma tissues arising in a female MMTV-PyVT/FVB transgenic mouse. Then this tumor tissue was implanted into the mammary fat pad (MFP) of syngeneic FVB/N mice. The MFP tumor was resected when the tumor reached a size of ~ 500 mm3. All the mice developed breast cancer lung metastases. This was followed by 3 serial in vivo selections of tumor metastases to the lungs from this tumor-MFP-implantation. Finally, a fresh metastatic lesion from the lung was obtained and cultured in vitro as a long-term passage monolayer cell line.** Figure 2**. Repeat experiments of MCa-M3C orthotopic tumor response to combined losartan with single dose radiotherapy. (A) MCa-M3C orthotopic tumors growth and response curves of control, losartan alone, 20 Gy local irradiation alone (S-IR), and combined losartan and irradiation therapy (Los+S-IR); Los indicates the start losartan treatment; and RT indicates single dose irradiation. (B) Mean tumor growth time (Days) for tumor to reach a mean volume of 500 mm3. Results of repeated experiments once again showed that S-IR or Los+S-IR treatment significantly delayed tumor growth compared to control, or losartan alone (S-IR vs Control/Losartan; and Los+S-IR vs Control/Losartan, all P<0.0001). In addition, the combination of Losartan+S-IR significantly enhanced tumor response compared to radiation alone (S-IR) by increasing TGD with 8 additional days (Los+S-IR vs S-IR, P<0.0001). However, losartan alone also did not show any effect on MCa-M3C tumors (Losartan vs Control, P>0.05). Data are presented as Mean ± SEM; P<0.05 is considered statistically significant (N=17-18/group).** Figure 3**. Tumor vessel imaging of MCa-M3C isografts in the MFP windows by MPLSM. (A) Tumor in a mouse treated with sterile water as control at day 0 and (B) day 3; and (C) tumor in a mouse treated with losartan at day 0, and (D) day 3. Bar=250 μm. (E) Gross pictures of lung metastases in control, Losartan, IR, and IR+Losartan treatment groups. **Figure 4**. H&E staining sections of losartan treated and control MCa-M3C MFP tumor tissues. (A) Tumor in a mouse treated with sterile water as control at day 3; and (B) tumor in a mouse treated with losartan at day 3 with morphologically observed tumor stroma vessels (*). Bar=100 μm.

## Data Availability

The datasets generated and/or analyzed during the current study are available from the corresponding author upon request.

## Abbreviations

BC: breast cancer
HER2: human epidermal growth factor receptor 2
BW: body weight
IR: irradiation
TGD: tumor-growth delay
AT1: angiotensin II receptor type 1
DMEM: Dulbecco’s modified Eagle’s medium
FBS: fetal bovine serum
PE: plating efficiency
SF: surviving fraction
CDT: cell doubling time
DAB: diaminobenzidine
MFP: mammary fat pad
ECM: extracellular matrix
CAFs: cancer-associated fibroblasts
