# Supplementary material for: Combining losartan with radiotherapy increases tumor control and inhibits lung metastases from a HER2/neu-positive orthotopic breast cancer model
Source: Radiat Oncol. 2021 Mar 4;16:48. doi: 10.1186/s13014-021-01775-9 (PMC7934382; doi:10.1186/s13014-021-01775-9)

Supplemental Figure 1

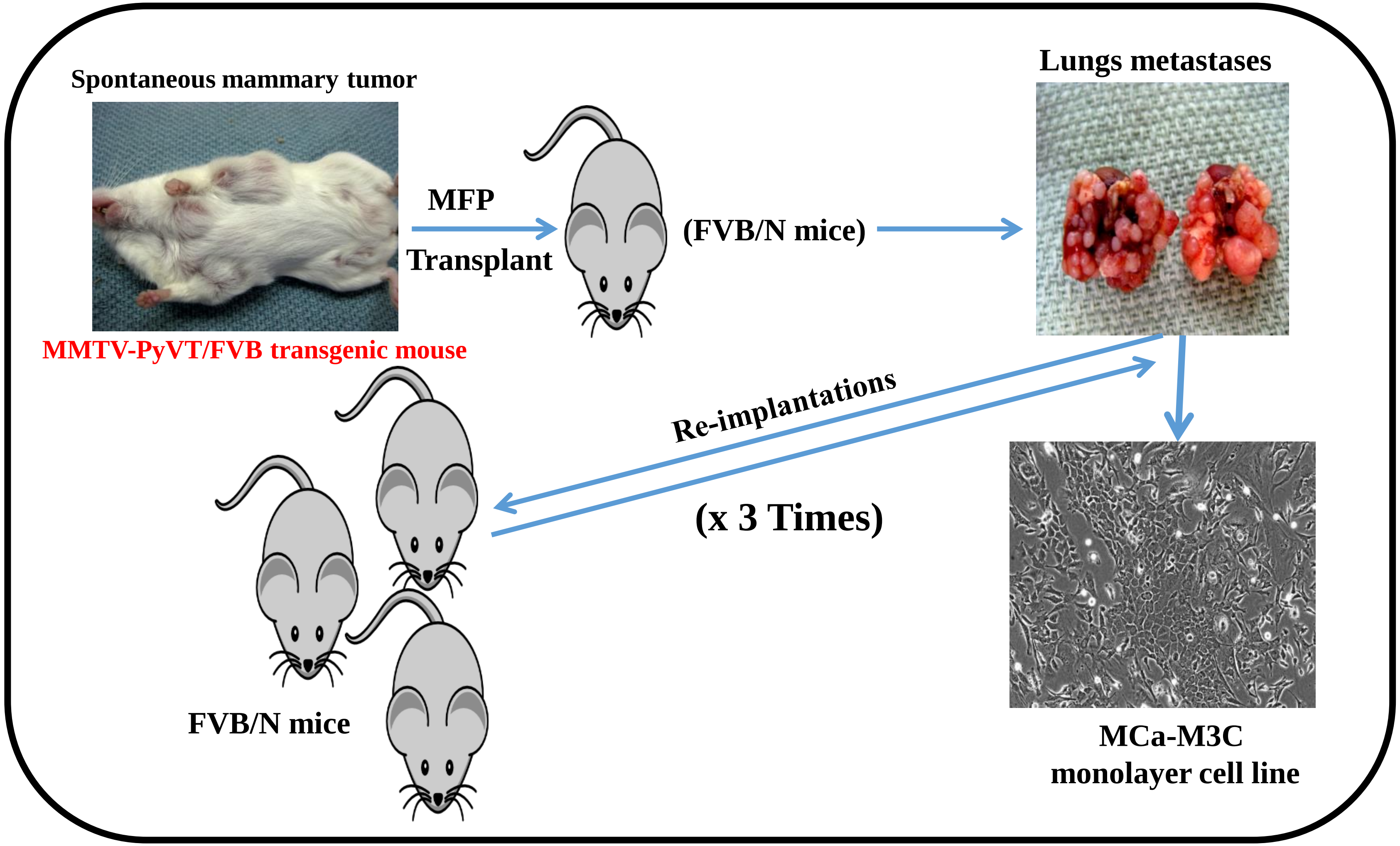

Supplemental Figure 2

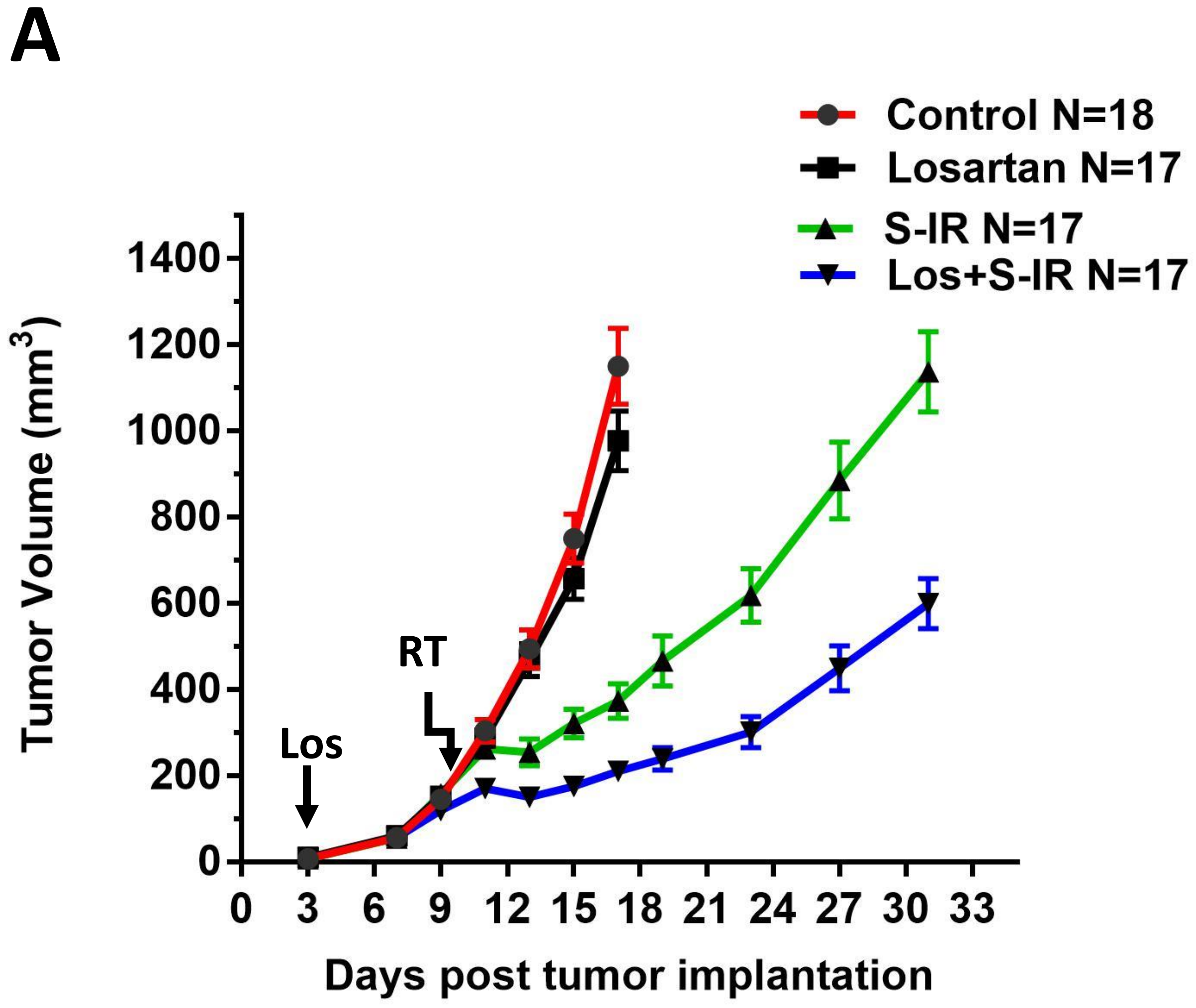

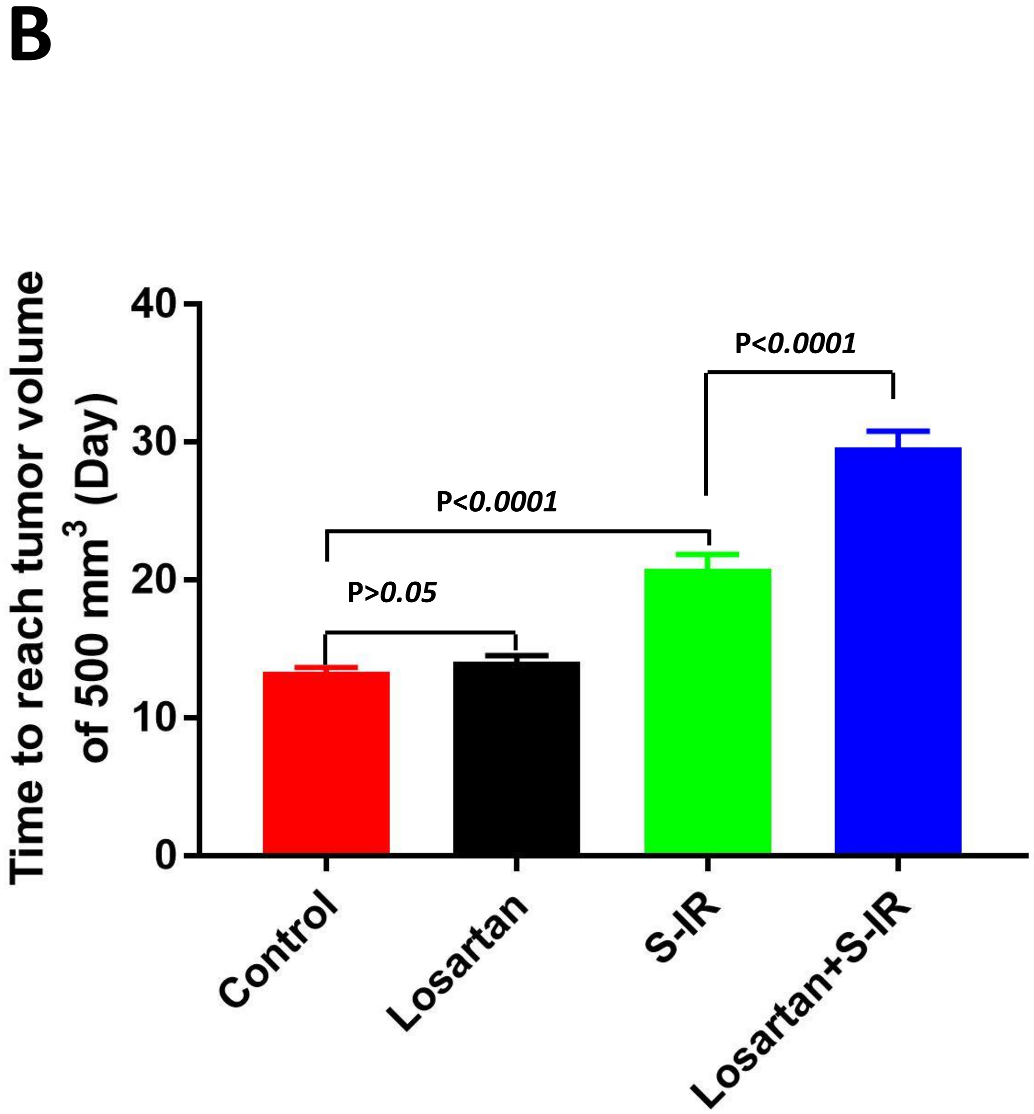

Supplemental Figure 3

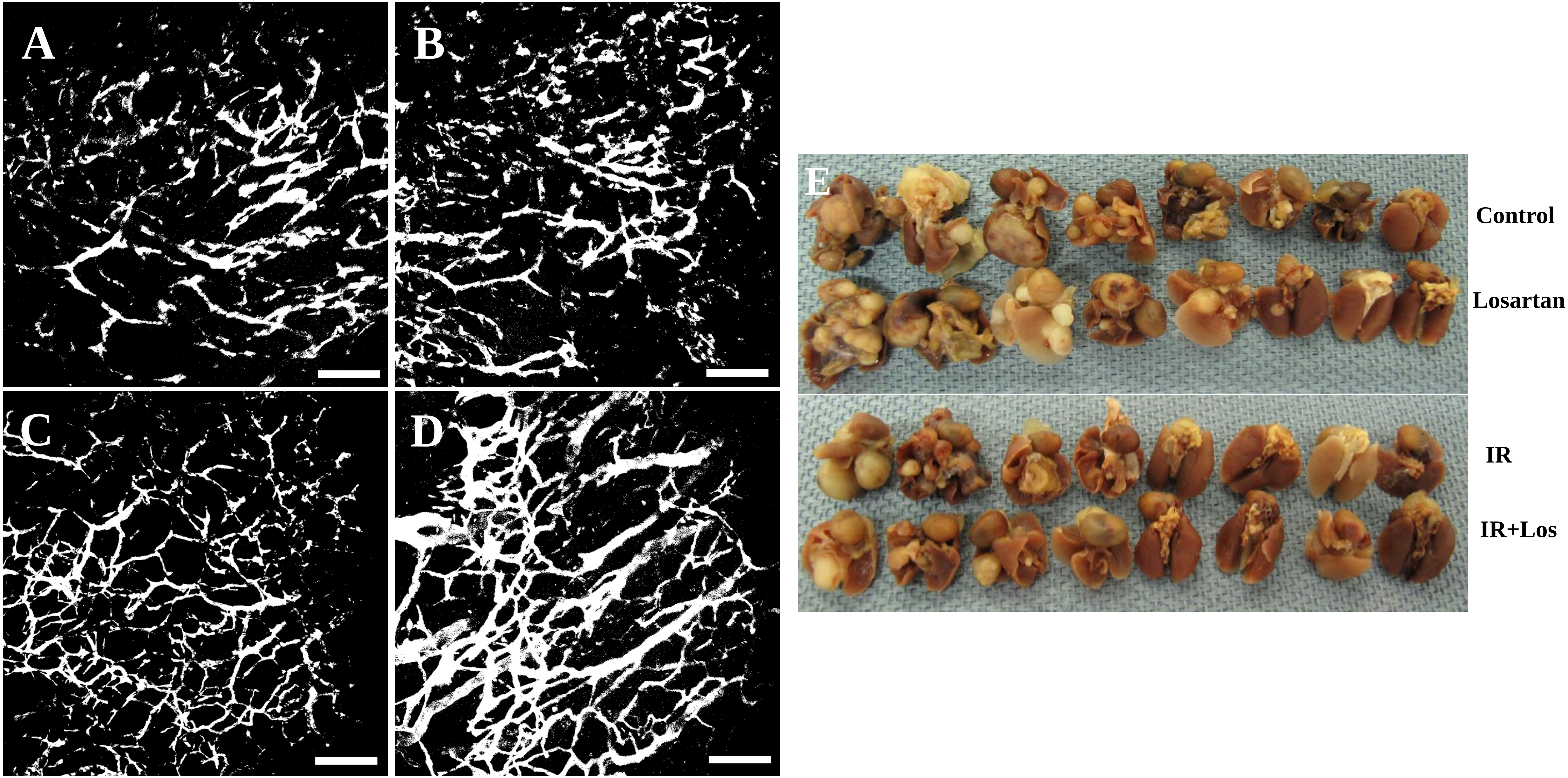

Supplemental Figure 4

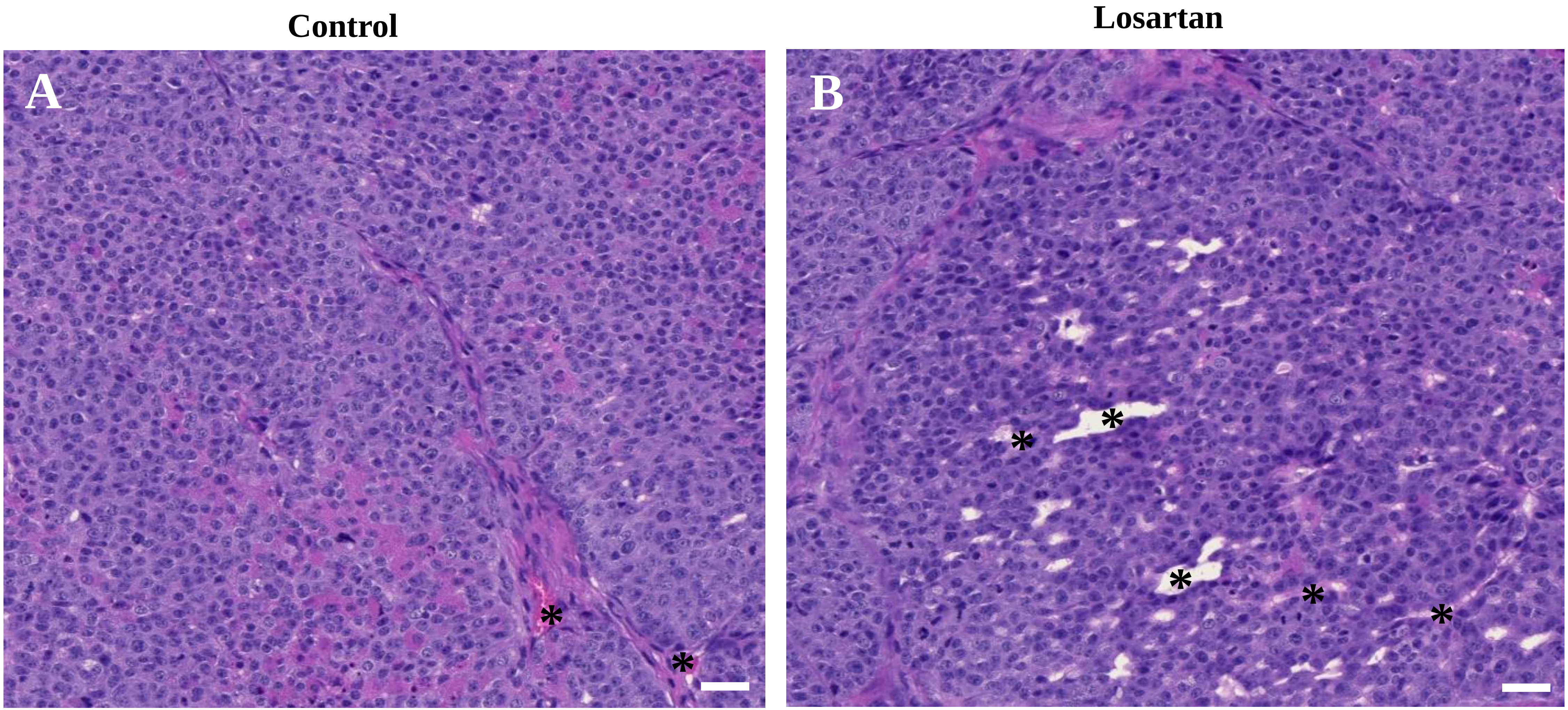

Supplement: Supplementary file 1 — Additional file 1. Figure 1. Schematic representation of the protocol for MCa-M3C cell line establishment. Tumor tissue was obtained from a spontaneous mammary adenocarcinoma tissues arising in a female MMTV-PyVT/FVB transgenic mouse. Then this tumor tissue was implanted into the mammary fat pad (MFP) of syngeneic FVB/N mice. The MFP tumor was resected when the tumor reached a size of ~ 500 mm3. All the mice developed breast cancer lung metastases. This was followed by 3 serial in vivo selections of tumor metastases to the lungs from this tumor-MFP-implantation. Finally, a fresh metastatic lesion from the lung was obtained and cultured in vitro as a long-term passage monolayer cell line. Figure 2. Repeat experiments of MCa-M3C orthotopic tumor response to combined losartan with single dose radiotherapy. (A) MCa-M3C orthotopic tumors growth and response curves of control, losartan alone, 20 Gy local irradiation alone (S-IR), and combined losartan and irradiation therapy (Los+S-IR); Los indicates the start losartan treatment; and RT indicates single dose irradiation. (B) Mean tumor growth time (Days) for tumor to reach a mean volume of 500 mm3. Results of repeated experiments once again showed that S-IR or Los+S-IR treatment significantly delayed tumor growth compared to control, or losartan alone (S-IR vs Control/Losartan; and Los+S-IR vs Control/Losartan, all P<0.0001). In addition, the combination of Losartan+S-IR significantly enhanced tumor response compared to radiation alone (S-IR) by increasing TGD with 8 additional days (Los+S-IR vs S-IR, P<0.0001). However, losartan alone also did not show any effect on MCa-M3C tumors (Losartan vs Control, P>0.05). Data are presented as Mean ± SEM; P<0.05 is considered statistically significant (N=17-18/group). Figure 3. Tumor vessel imaging of MCa-M3C isografts in the MFP windows by MPLSM. (A) Tumor in a mouse treated with sterile water as control at day 0 and (B) day 3; and (C) tumor in a mouse treated with losartan at day [file 13014_2021_1775_MOESM1_ESM.pdf]
